# Carbon Monoxide-Releasing Molecule-2 Reduces Intestinal Epithelial Tight-Junction Damage and Mortality in Septic Rats

**DOI:** 10.1371/journal.pone.0145988

**Published:** 2015-12-31

**Authors:** Shulong Zhang, Shuyun Zheng, Xin Wang, Qiankun Shi, Xiang Wang, Shoutao Yuan, Guozheng Wang, Zhenling Ji

**Affiliations:** 1 Department of General Surgery, Zhongda Hospital, Southeast University Medical School, Nanjing, Jiangsu 210009, China; 2 Department of Critical Care Medicine, Nanjing First Hospital, Nanjing Medical University, Nanjing, Jiangsu 210006, China; 3 Institute of Infection and Global Health, University of Liverpool, Liverpool, L69 7BE, United Kingdom; Aix-Marseille University, FRANCE

## Abstract

**Objective:**

Damage to intestinal epithelial tight junctions plays an important role in sepsis. Recently we found that Carbon Monoxide-Releasing Molecule-2 (CORM-2) is able to protect LPS-induced intestinal epithelial tight junction damage and in this study we will investigate if CORM-2 could protect intestinal epithelial tight junctions in the rat cecal ligation and puncture (CLP) model.

**Materials and Methods:**

The CLP model was generated using male Sprague-Dawley (SD) rats according to standard procedure and treated with CORM-2 or inactive CORM-2 (iCORM-2), 8 mg/kg, i.v. immediately after CLP induction and euthanized after 24h or 72h (for mortality rate only). Morphological changes were investigated using both transmission electron and confocal microscopy. The levels of important TJ proteins and phosphorylation of myosin light chain (MLC) were examined using Western blotting. Cytokines, IL-1β and TNF-α were measured using ELISA kits. The overall intestinal epithelial permeability was evaluated using FD-4 as a marker.

**Results:**

CORM-2, but not iCORM-2, significantly reduced sepsis-induced damage of intestinal mucosa (including TJ disruption), TJ protein reduction (including zonula occludens-l (ZO-1), claudin-1 and occludin), MLC phosphorylation and proinflammatory cytokine release. The overall outcomes showed that CORM-2 suppressed sepsis-induced intestinal epithelial permeability changes and reduced mortality rate of those septic rats.

**Conclusions:**

Our data strongly suggest that CORM-2 could be a potential therapeutic reagent for sepsis by suppressing inflammation, restoring intestinal epithelial barrier and reducing mortality.

## Introduction

Sepsis is a systemic inflammatory syndrome with oxidative damage, coagulation disorders and tissue hypoperfusion and may be companied with immune suppression, and severe intestinal mucosal barrier dysfunction[1]. The intestinal mucosal barrier can prevent intestinal flora, which contains endogenous and exogenous microorganisms, from becoming pathogens of sepsis under certain circumstances by preventing flora from crossing into the body via both transcellular and paracellular pathways[2]. Tight junction (TJ) proteins are key molecules for determination of the paracellular permeability[3]. In some studies of intestinal inflammatory diseases, the intestinal mucosal barrier is compromised with decreased expression and differential distribution of tight junction proteins [4–6]. Therefore, novel interventions targeting intestinal mucosal barrier dysfunction may reduce unfavorable inflammatory responses to improve survival during sepsis.

One of the main metabolites of heme degradation by heme oxygenase-1 (HO-1) [7,8] is endogenous carbon monoxide (CO), which was shown to be protective against the symptoms of ulcerative colitis, and this offers a plausible correlation for the beneficial effects of CO in reducing intestinal mucosal permeability [9]. By investigating its vasoactive, anti-inflammatory, anti-apoptotic, and cytoprotective properties in various models of disease, the possibility that CO could be used clinically was proposed [10–12]. The protective effects of CO have been shown in septic models [13]. Recently, a new class of CO-releasing molecule-2 (CORM-2), which releases CO to the tissues, has been synthesized [14] with therapeutic potential for diseases like sepsis [13].

We reported that CORM-2 in vitro protects the intestinal epithelial barrier through reducing pro-inflammatory cytokines and increasing TJs protein expression. To explore the therapeutic potential of CORM-2 in sepsis, a rat CLP model is selected due to its similarity to human peritonitis-sepsis and the reliability of the model[15]. As far as we are aware, the role of CORM-2 in protecting the intestinal epithelial tight junctions (IETJs) in septic rats has not been reported. This study aims to investigate whether CORM-2 can release CO to regulate the levels of IETJs proteins, protect intestinal epithelial barrier function and eventually improve survival in septic rats in order to lay a foundation for future development of new therapy for patients with sepsis.

## Materials and Methods

### Reagents

CORM-2 from Sigma (St Louis, MO. USA) was dissolved in dimethyl sulfoxide (DMSO) to obtain a 40 mM stock with inactive form (iCORM-2) of the compound prepared as described previously [16] and used as a negative control in some experiments. Anti-zonula occludens protein-1 (ZO-1), claudin-1 and occludin from Santz Cruz Biotechnology (Santa Cruz, CA); anti-total-myosin light chain (MLC) and anti-phospho-MLC (p-MLC) from Cell Signaling Technology (Danvers, MA, USA); anti-β-actin from KeyGen BioTECH (Nanjing, China) were used in this study. Enzyme-linked immunosorbent assay (ELISA) kits from Hermes Criterion Biotechnology (HCB, Vancouver, Canada) was used.

### Animals and sepsis model

The guidelines for the care and use of laboratory animals published by the National Institute of health (NIH) were followed throughout the experiments. Experimental protocols were approved by the Nanjing Medical University Committee on Animal Care and all the works have been done under the license of SYXK (Jiangsu province, No. 2009–0015) by well-trained researchers. Male Sprague-Dawley (SD) rats (8–10 weeks old with body weights of 220–260g) from the Animal Center of Nanjing Medical University (Nanjing, China) were kept in a temperature controlled room with a 12h light/12h dark cycle for 1 week before the experiment, with free access to food and water. CLP model of were used because this is a best model to mimic human peritonitis-sepsis and all procedures in rats are easier and more reliable than in mice.

### Experimental design and sample sizes

The rats were divided into 4 groups based on a random number table to reduce bias: the cecal ligation and puncture (CLP) group, the CLP + CORM-2 (CORM) and CLP + iCORM-2 (iCORM) groups (8 mg/kg CORM-2 and iCORM-2, i.v. respectively) [17], and the sham group with a sham operation without CLP. We used power analysis, with a power of 80%, to determine the sample sizes required in each experimental group using values that have been determined in a preliminary dose response experiment using a minimum number of rats. For investigation of the pathological changes and cytokine release, minimum 10 rats per group were required and for measuring intestinal permeability, minimum 5 rats per group were required. For comparison, all rats were euthanized at 24 h after CLP procedure by dislocating neck after sedated by i.p. injection of (300 mg/kg) 10% chloral hydrate. For mortality rates, a minimum of 12 rats were required. After treatment, the animals had free access to food and water and were monitored every 3 hours for 72 h to record survival rates. Any rat that showed the signs of suffering was given a low dose of chloral hydrate (i.p 50–100 mg/kg) and kept under continuous monitoring of physical appearance, such as posture, eating and drinking, urine, skin and fur, abdominal enlargement or ascites, measuring breath and heart rates, body weight and temperature every 2 h, and checking mobility, unprovoked behavior and response to external stimuli on regular basis. The dying rat (judged by lack of mobility and no response to stimuli) was euthanized by cervical dislocation as described above; otherwise at 72 h. Rats could not be reused in these 3 sets of experiments. No unexpected death occurred in this study.

### Generation of CLP model

One dose of urethane (1.5 g/kg) and ketamine (50 mg/kg) was injected subcutaneously for general anesthesia. On the anterior abdomen, a 2-cm midline incision was made to expose cecum, which was ligated just distal to the ileocecal valve to avoid any intestinal obstruction. The isolated cecum was punctured twice with a sterile 20-gauge needle and squeezed to expel a small amount of fecal material. Then the abdomen was then closed using atraumatic silk sutures. The same surgical procedure was performed on sham-operated animals except that the cecum was neither ligated nor punctured. Two groups of CLP rats were treated with 8 mg/kg CORM-2 or iCORM-2 through tail veins 2 h after CLP procedure. The sham group and CLP alone group were treated with saline i.v. as controls. All animals also received normal saline (5ml/100g) subcutaneously immediately after the operation to produce a hyperdynamic phase to mimic early stages of sepsis in this experimental model [18].

### Measurement of intestinal permeability

Intestinal permeability was studied as described previously [19] and some modification was made. Rats were anesthetized and the distal intestine (a 10 cm long segment from 5cm up the ileocecal valve) was ligated to make a closed loop. The blood supply of this section of ileum was not disturbed. Fluorescein isothiocyanate (FITC)-labeled dextran (FD-4) solution (20 mg/ml, 1ml) was injected into the loop. Blood was withdrawn from the portal vein after 30 min. Then serum was isolated and the concentration of FD-4 was determined by a fluorescence spectrophotometer at an excitation wavelength of 490 nm and an emission wavelength of 520 nm.

### Serum cytokine analysis

Blood samples were taken from the portal vein and isolated sera were stored at −80°C until analyzed. Concentrations of TNF-α and IL-1β were determined by ELISA kits.

### Histopathological examination

The intestines of the septic and sham-operated rats were harvested at 24 h after CLP and fixed in 4% paraformaldehyde. After being embedded in paraffin the tissues were sectioned and then stained with the hematoxylin and eosin (HE) reagent according to standard protocols. The images were taken using light microscopy (×200 magnification) and intestinal mucosal injury was evaluated by the Chiu scoring system [20]. Ten fields for each sample were examined, and the average scores were calculated.

### Transmission electron microscopy

The intestines from 10 rats per group were fixed and analyzed by transmission electron microscopy (TEM) as follows. Pieces of ileum, about 1 mm^3^, were fixed with 2.5% glutaraldehyde overnight at 4°C and then incubated for 2 h with 1% osmium tetroxide and finally dehydrated through a graded series of acetone. The fixed tissues were embedded in epoxy resin and sectioned (75 nm). The sections were then stained with uranyl acetate and lead citrate and examined under an H-600 Electron Microscope (JEM 1010, Hitachi, Japan) at 80 kV. TJ lengths were measured using images that oriented in parallel to the axis of microvilli by two qualified pathologists who were blinded to the experiments.

### Immunofluorescence microscopy

Ten rats per group were analyzed by immunofluorescence. The ileum segments were immediately removed after euthanization and stored at -80°C after being snap-frozen in the embedding medium (Tissue-Tek, O.C.T. Compound, Sakura). The frozen ileum was cut into 4μm thick sections which were fixed with cold acetone for 10 min at 4°C, and blocked with 5% fetal bovine serum in PBS. The sections were then incubated with anti-ZO-1 (1:100); anti-occludin (1:100); or anti-claudin-1 (1:100) overnight followed by FITC-conjugated and Cy3-conjugated secondary antibodies. After mounting, the slides were examined under a laser confocal microscope (Olympus FV10i, Japan).

### Western blotting

Ten rats per group were analyzed by Western blotting. For Western blotting, scrapped ileum mucosa samples were snap frozen and store at −80°C until further use. Ileum mucosa samples were homogenized with an automatic homogeniser in chilled SDS-lysis buffer. After centrifugation, the protein concentration in supernatant was determined using a BCA protein assay kit (Beyotime, China) and 50 μg per sample were subjected to SDS-PAGE and Western blotting. Intensities of immunoreactive bands were quantified using Gel-Pro Analyzer 4.0 (MediaCybernetics, USA). The target proteins/β-actin ratios were calculated for comparison.

### Statistical analysis

Data were represented as means±SD and assessed by ANOVA followed by Newman-Keuls test. Mortality was analyzed using Kaplan-Meier survival curves, and then log-rank test for statistical differences in survival rates. SPSS 19.0 (SPSS Inc., Chicago, IL, USA) were used for those analysis.

## Results

### CORM-2 improves morphological changes of intestinal mucosa during sepsis

**Fig 1:**
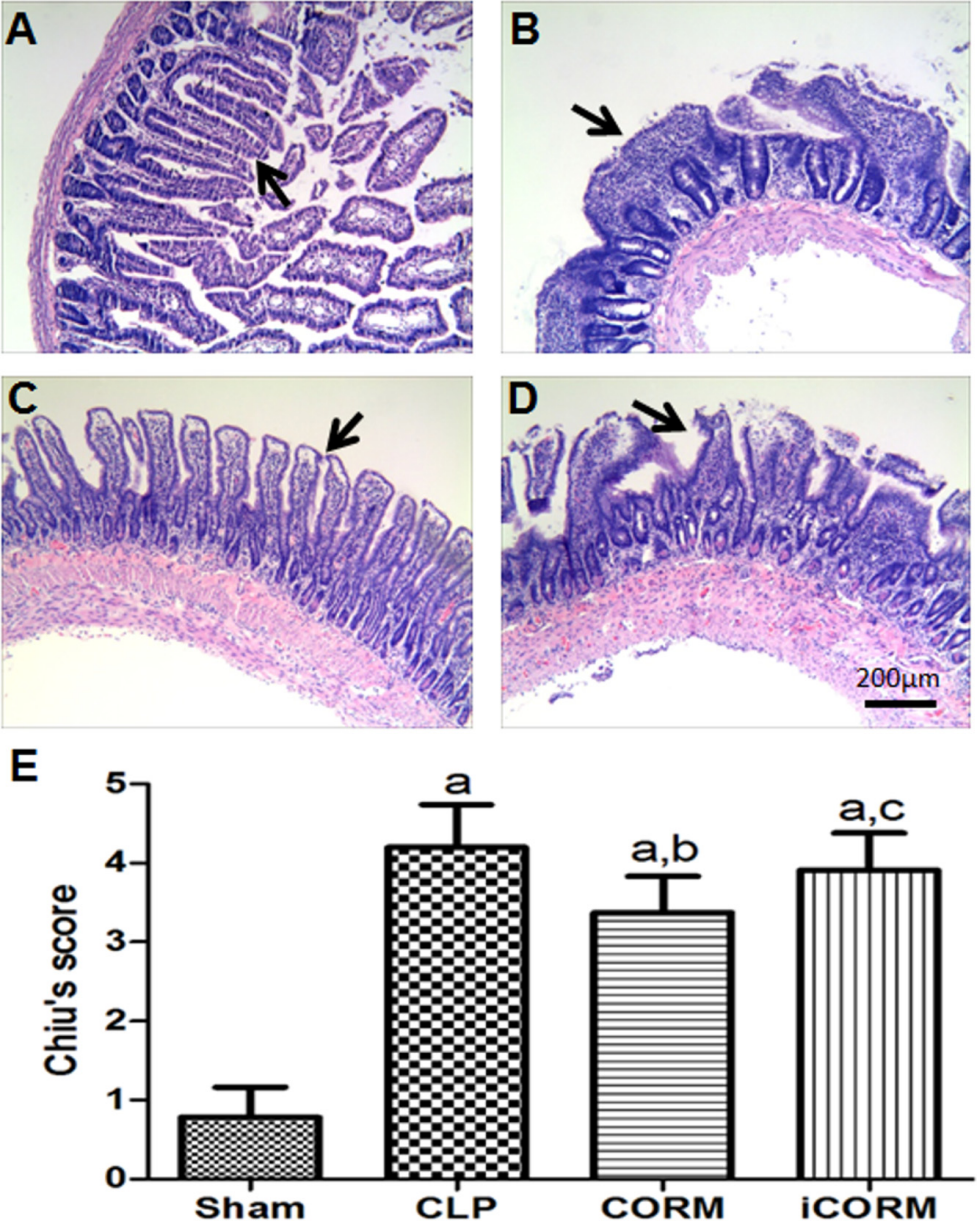
Effect of CORM-2 on CLP-induced intestinal inflammation. Typical images of H&E stained intestinal sections from sham (A), CLP (B) rats or CLP rats with CORM-2 (C) or iCORM-2 (D) treatment. Severe inflammation (ulceration, hemorrhage and epithelial loss) was found in CLP rat (B) and CLP rats treated with iCORM (D). These changes were alleviated CLP rats treated with CORM-2 (C). These differences were semi-quantified using Chiu score system of mucosal injury by 2 investigators (E). Data are shown as means±SD. ^a^P<0.05, compare to Sham rats; ^b^P<0.05, compare to CLP rats; ^c^P<0.05, compare to CLP rats treated with iCORM-2. Bar = 200μm.

In the sham group, the structure of the small intestinal mucosa was intact, and normal intestinal mucosa was observed (Fig 1A). Severe mucosal injury was found in CLP alone, CORM-2 and iCORM-2 treated CLP rats (Fig 1B–1D). However, rats with CLP alone (Fig 1B) and CLP rats treated with iCORM-2 (Fig 1D) displayed more severe mucosal injury compared to CORM-2 treated CLP rats (Fig 1C). Using the Chiu’s score system, we semi-quantified the lesions in all groups (40/40 rats) and found that CLP rats (4.2±0.54) have significantly higher scores than the sham group (0.78±0.39) (P<0.05), but in CORM-2 treated CLP rats (3.37±0.46), the injury was significantly reduced compared to CLP alone or treated with iCORM-2 (3.91±0.47) (P<0.05) (Fig 1E). However, the scores of iCORM-2 treated CLP rats is slight lower than that of CLP rats without treatment but there was no statistical significance. These data suggest that CORM-2 could partially reduce the CLP-induced intestinal mucosa injury.

### Effects of CORM-2 on sepsis-induced TJ disruption

**Fig 2:**
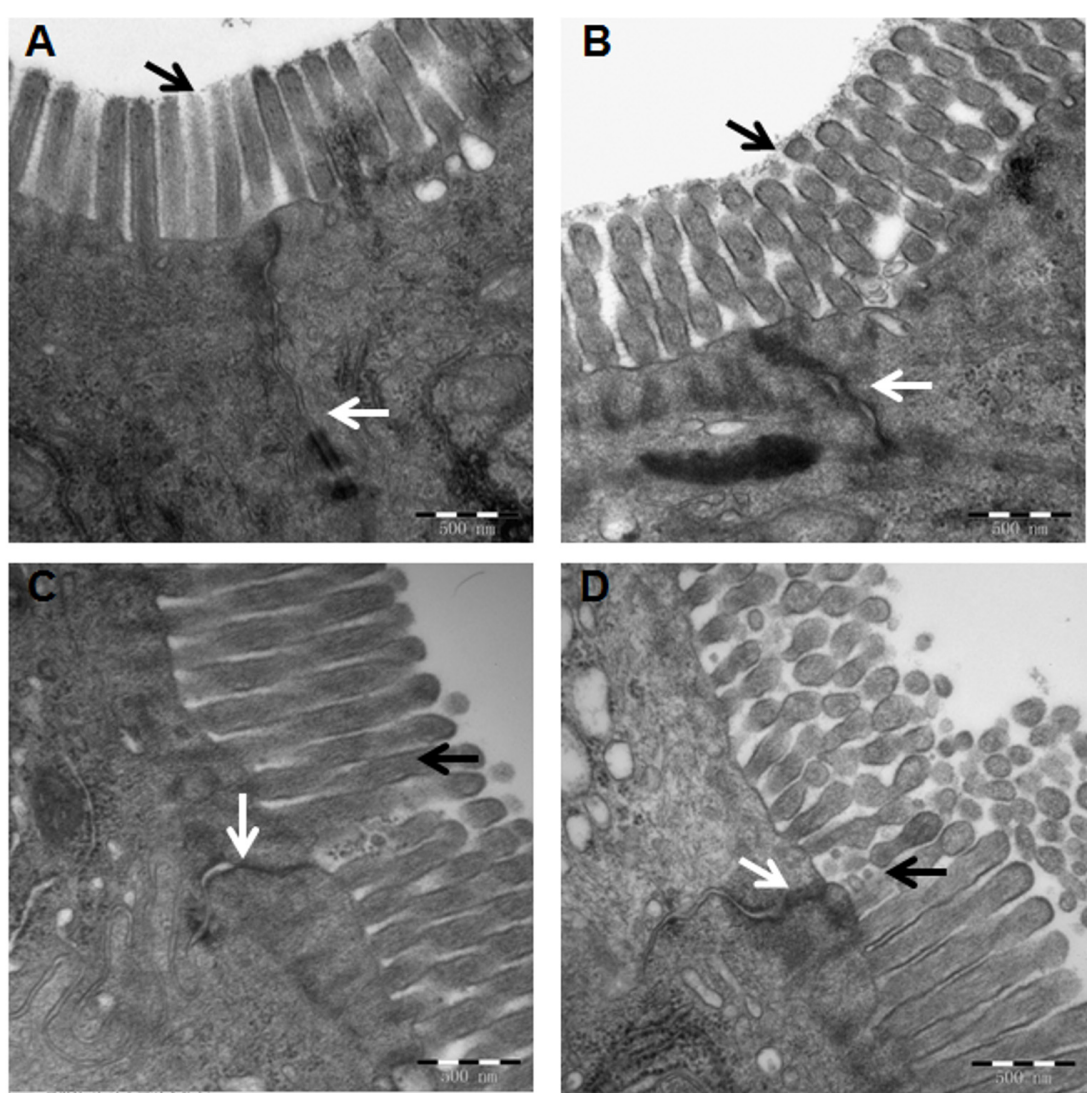
Tight junctions of intestinal epithelium were revealed by transmission electronic microscopy. Typical images from Sham rats (A), CLP rats (B), CLP rats treated with CORM-2 (C) or iCORM-2 (D). Black arrows indicate epithelial cell surface microvilli and white arrows indicate tight junctions. Magnification × 60000.

Since TJs play critical roles in the maintaining intestinal mucosa barrier permeability, the ultrastructural changes of intercellular TJs were examined by transmission electron microscopy. The TJs of 20 out of 40 rats, i.e. 5 rats per group were examined due to the extensive labor required for processing and examining each sample. In the sham group, the rows of epithelial cells were arranged closely, and epithelial cell surface microvilli were arranged in neat rows (Fig 2A). The TJ stand and desmosome were clear and complete, and the paracellular spaces were narrow (Fig 2A). In the CLP group, the microvilli were sparse with irregular length and arrangement (Fig 2B). The TJ stand and desmosome were obscured or had disappeared, and the paracellular spaces were wider (Fig 2B). CORM-2 treatment obviously improved CLP-induced TJ disruption (Fig 2C) with respect to structural integration and increased the closeness of intercellular connection. In contrast, no obvious improvement by iCORM-2 treatment was observed (Fig 2D). These results demonstrated that CORM-2 could reduce the TJ ultrastructure distortion induced by sepsis.

### CORM-2 suppressed the disarrangement of TJ proteins in septic rats

**Fig 3:**
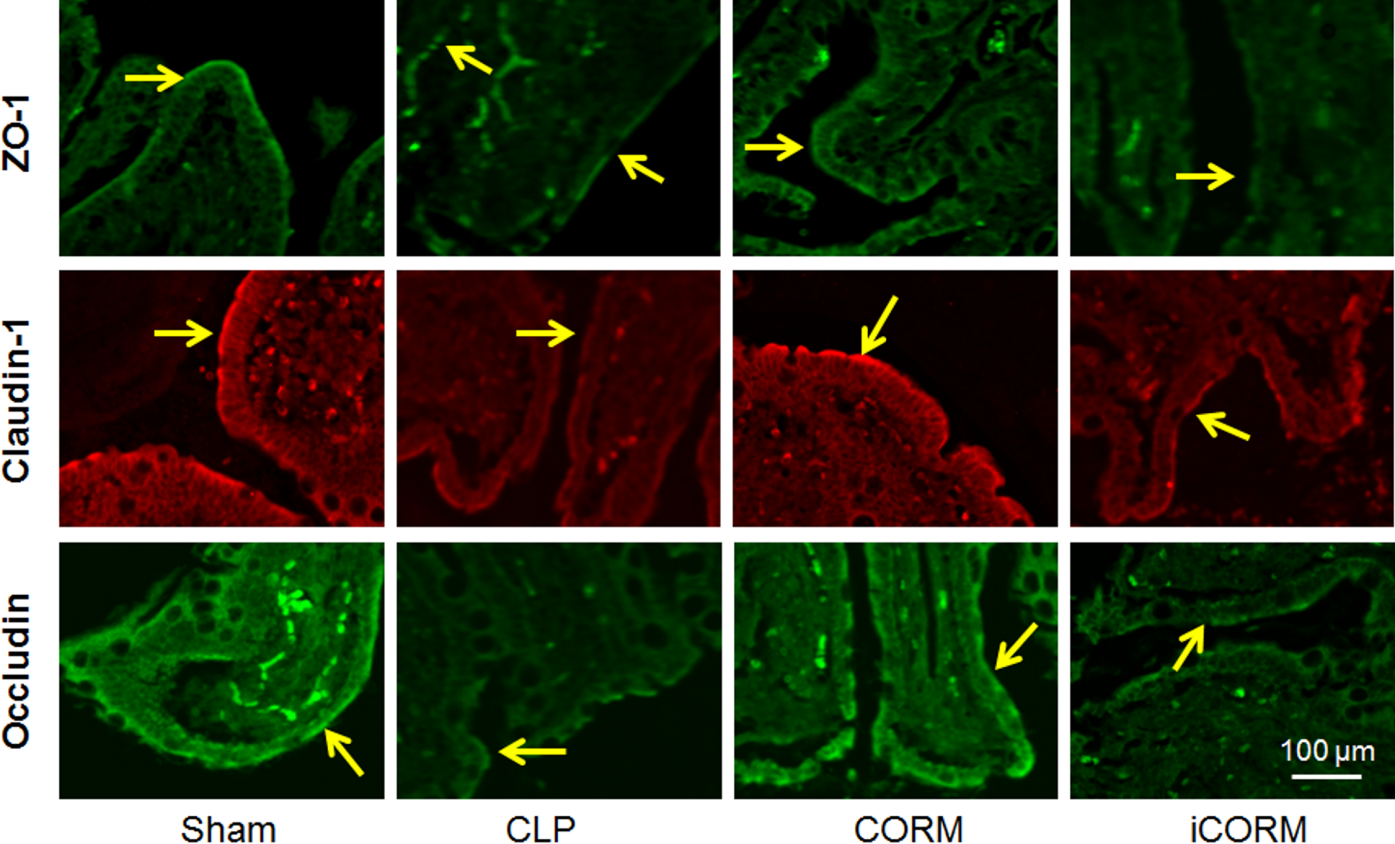
Effects of CLP and CORM-2 on tight junction proteins and intestinal epithelial barriers. Anti- ZO-1, claudin-1 or occludin were used to stain the sections of ileum from different groups of rats. Typical images were presented. Arrows indicate the highlighted intestinal epithelial barriers. Scale bars = 100μm.

Indirect immunofluorescence staining for ZO-1, claudin-1 and occludin was performed (40/40 rats) (Fig 3). In the sham group, ZO-1, claudin-1 and occludin staining was found at the apical part of the lateral membranes of the polar epithelial cells and distributed continuously to highlight an intact barrier under confocal microscope. In the CLP Group the signals were intermittent and markedly weaker than those in the Sham group showing that the integrity of the barrier was disrupted. CORM-2 treatment significantly reduced the disruption, but the iCORM-2 treatment did produce any obvious changes.

### CORM-2 alleviates the reduction of TJ proteins induced by sepsis

**Fig 4:**
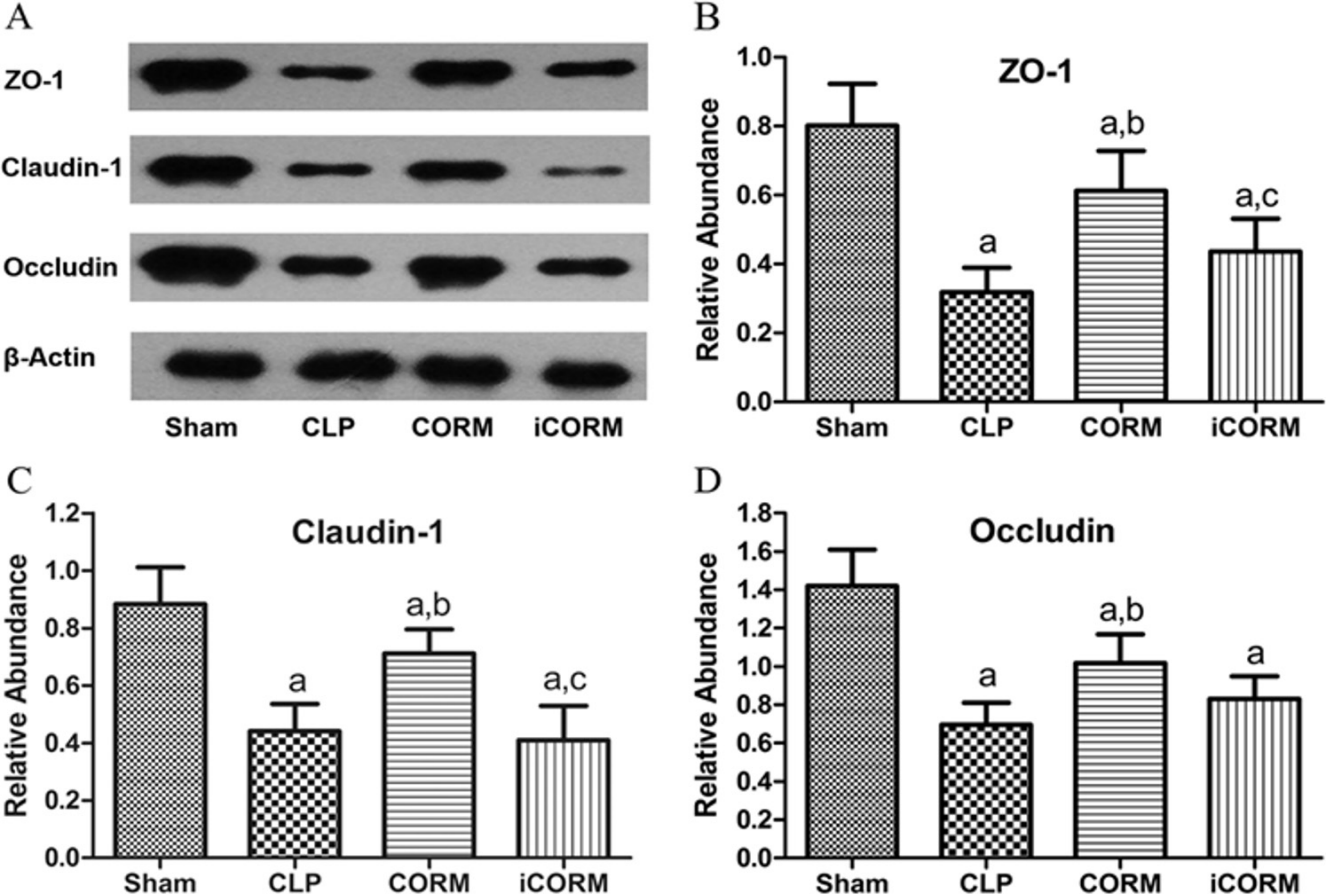
Effects of CLP and CORM-2 on the expression levels of tight junction proteins. Ileum from different groups of rats were collected and homogenized. The tissue lysates were subjected to Western blotting analysis using specific antibodies for ZO-1, claudin-1 or occludin with β-actin as an endogenous control. Typical Western blots are presented (A). The band intensity was quantified using densitometry. The means±SDs of ZO-1/β-actin ratios (B); claudin-1/β-actin ratios (C); and occludin/β-actin ratios (D) from different groups of rats are presented. ^a^P < 0.05 compared to Sham rats; ^b^P < 0.05 compared to CLP rats; ^c^P < 0.05 compared to CLP rats treated with CORM-2.

Using Western blotting (40/40 rats), the levels of ZO-1, occludin, or claudin-1 in small intestines of all CLP rats (with or without treatment) were significantly reduced compared to sham operation rats (P<0.05) (Fig 4). However, CORM-2 treatment increased the expressions of occludin, claudin-1 and ZO-1 significantly (P<0.05) compared to that in CLP alone rats or CLP rats treated with iCORM except that there was no significant difference for occludin. These data showed that CORM-2, but not iCORM-2, increased certain TJ proteins in CLP rats.

### CORM-2 down-regulates myosin light chain (MLC) phosphorylation in intestinal mucosa of septic rats

**Fig 5:**
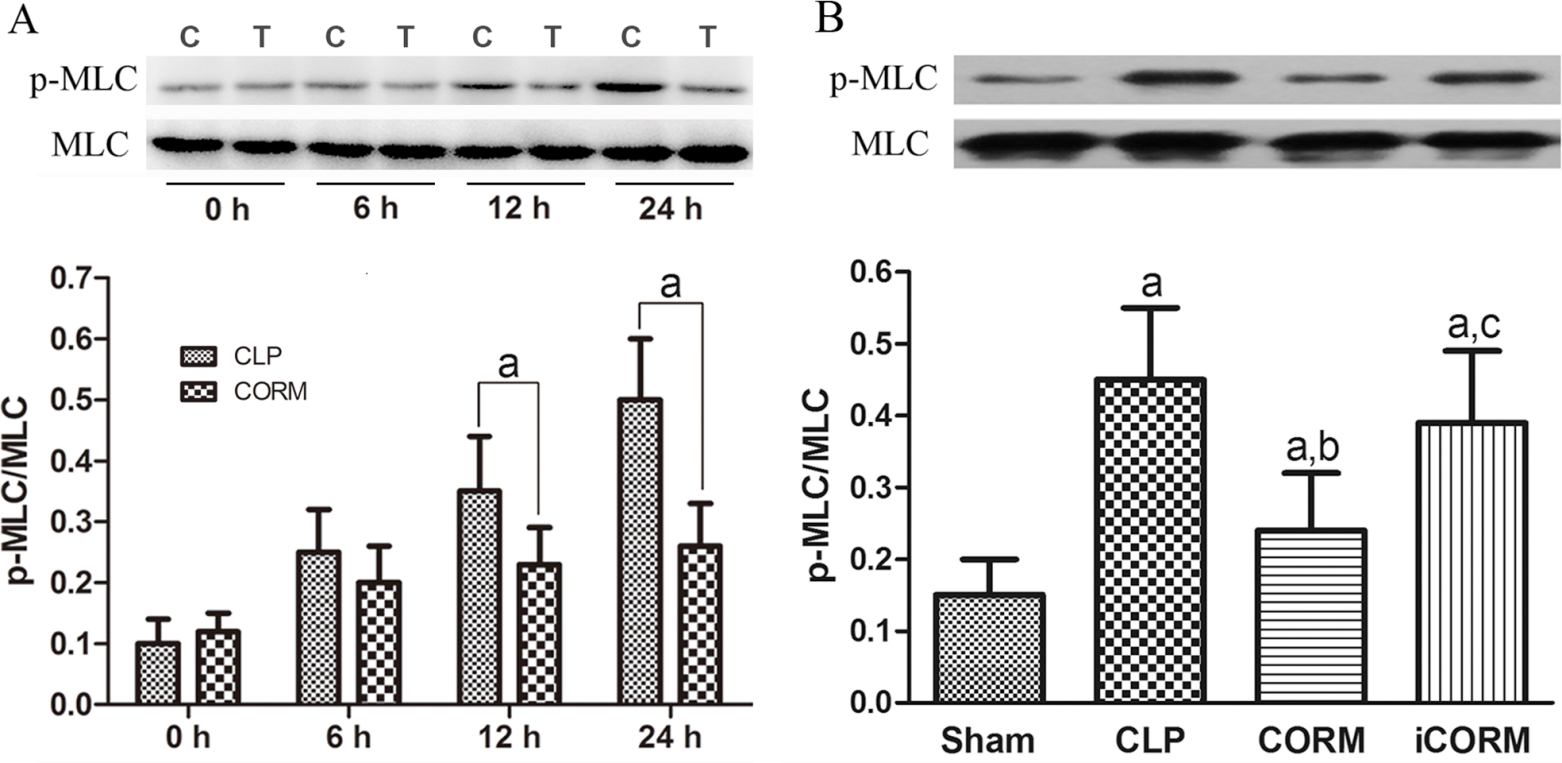
Effects of CLP and CORM-2 on MLC phosphorylation in rat ileum. The lysates of ileum from different groups of rats were subjected to Western blotting analysis with anti-MLC and phosphorylation-specific antibody for p-MLC. Typical Western blots and Means±SD of the ratios of p-MLC/MLCcalculated based on the densities of bands on Western blots from four groups are presented. (A): Time course of MLC phosphorylation in CLP (C) and CORM-2-treated (T) CLP rats (5 rats each time point per group) ^a^P < 0.05 compared to CLP group. (B) The MLC phosphorylation levels at 24 h of each experimental group. ^a^P < 0.05 compared to Sham rats; ^b^P < 0.05 compared to CLP rats; ^c^P < 0.05 compared to CLP rats treated with CORM-2.

To investigate the mechanisms of CLP induced sepsis and the effects of CORM-2, the level of MLC phosphorylation in intestinal mucosa at different time points was examined using Western blotting with phosphorylation-specific antibodies (5 rats each time point). We found that the MLC phosphorylation levels significantly increased after 12 h in CLP rats. In contrast, in CORM-2 treated CLP rats, MLC phosphorylation was maintained in a basal level even at 24h (Fig 5A, up panel). The difference between CLP rats and CORM-2-treated CLP rats in MLC phosphorylation became significant at 12 h and 24 h, indicating the inhibitory effect of CORM-2. We further compared the levels of MLC phosphorylation at 24 h of each experimental group (10 rats each group). We found that in the sham group, the levels of phosphorylated MLC were low compared with the other three CLP groups. The CORM-2 treated but not iCORM-2 treated CLP rats showed significant reduction of MLC phosphorylation compared to CLP group. Those data demonstrated that CORM-2 could significantly attenuate CLP-induced MLC phosphorylation.

### CORM-2 alleviated the increase in TNF-α and IL-6 of CLP rats

**Fig 6:**
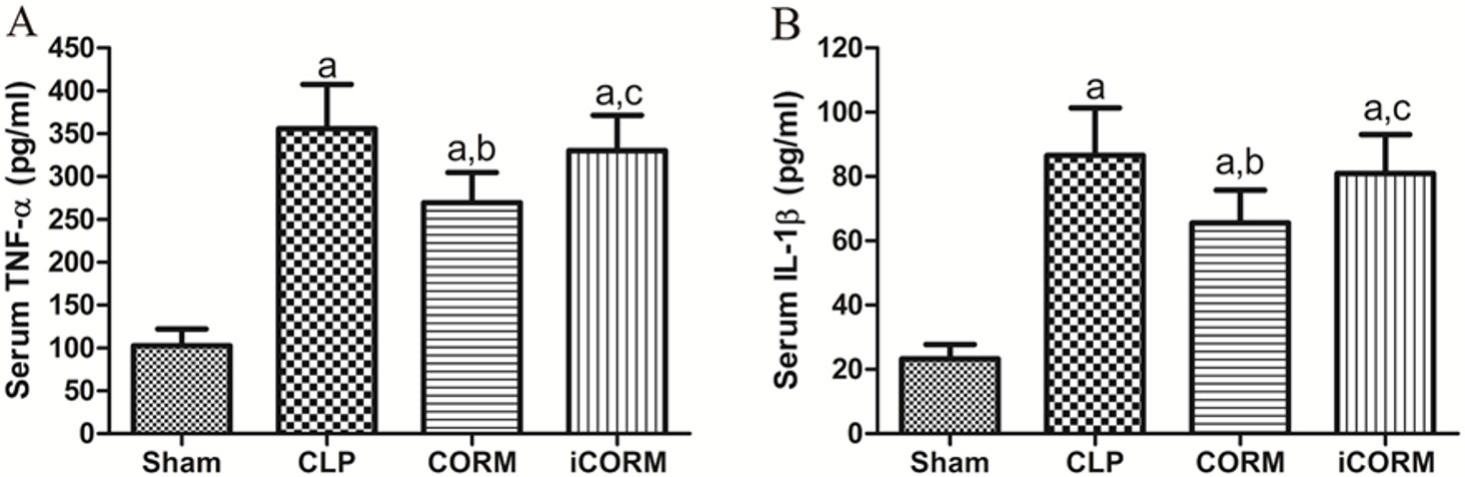
Effects of CORM-2 on CLP-induced proinflammatory cytokine release. Blood was taken from portal vein at 24h after sepsis induction by CLP. Concentrations of TNF-α and IL-1β were measured using ELISA kits. Means±SD from 10 rats per group are presented. ^a^P<0.05, compare to Sham rats; ^b^P<0.05, compare to CLP rats; ^c^P<0.05, compare to CLP rats treated with iCORM-2.

Blood samples from 40/40 rats were analyzed and means±SD is shown in Fig 6. Serum TNF-α and IL-1β were elevated in CLP rats compared to the sham control. CORM-2 treatment after CLP was able to significantly alleviate the elevation, which is consistent with our *in vitro* data. In contrast, the same dose of iCORM could not reduce the CLP-induced elevation of both TNF-α and IL-1β, indicating that CO released from CORM-2 has anti-inflammatory properties in vivo.

### CORM-2 decreases CLP-induced intestinal permeability changes and mortality

**Fig 7:**
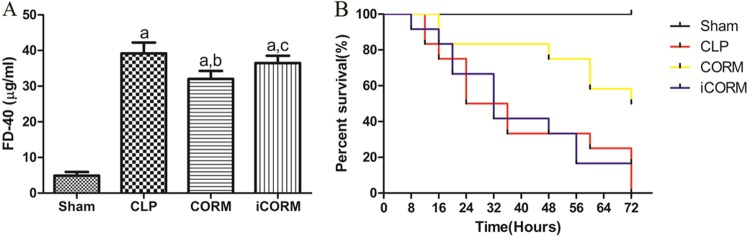
Effects of CORM-2 on intestinal permeability and survival of CLP rats. FD-4 was injected into a loop of intestine (See methods) and 30min late, the levels of FD-4 in portal vein were measured to reflect intestinal epithelial permeability changes. Means±SD from 5 rats each group are presented (A). Separated rats for each group (12 rats per group) were used to determine 72h mortality rates. Kaplan-Meier survival curves are shown (B). ^a^P < 0.05 compared to Sham rats; ^b^P < 0.05 compared to CLP rats; ^c^P < 0.05 compared to CLP rats treated with CORM-2.

Intestinal barrier function was evaluated by measuring the serum concentration of FD-4 (20/20 rats, Fig 7A). The serum concentration of FD-4 in the sham group (4.90±1.02) was very low compared to that in the CLP alone (39.17±3.05), CLP treated with CORM-2 (32.00±2.26) or iCORM-2 (36.47±2.06) (P<0.05). The levels of FD-4 were significantly decreased by CORM-2, but not iCORM-2 treatment (P<0.05) compared to CLP alone. No mortality was observed within 72h in sham rats. The survival rate after CLP alone was 50% (6/12) at 24h, and decreasing to 0% (0/12) at 72h (Fig 7B). However, CORM-2 treatment significantly increased the survival rate, over 80% (10/12) at 24h and 50% (6/12) at 72h (P<0.05). In contrast, iCORM-2 treatment did not increase survival rate (0/12 at 72h) post CLP. These data strongly indicate that CORM-2 could improve the function of intestinal epithelial barrier during sepsis and overall outcomes.

## Discussion

Despite the increased understanding of the pathophysiology of sepsis and the development of new treatments, sepsis is still the leading cause of death in intensive care units [21]. Previous studies have implicated that the alteration of intestinal barrier function plays a critical role in sepsis [22,23] and suggests that the gut might serve as the ‘‘motor” of the systemic inflammatory response syndrome [24]. As a consequence, increased intestinal permeability and intestinal barrier dysfunction indicate poor outcomes [25,26].

In this study, we demonstrate that CO released from CORM-2 could partly alleviate the intestinal epithelial tight-junction damage in CLP septic rats. In histopathological examination, the changes of Chiu’s scores of distal ileums demonstrate that CORM-2 is able to alleviate morphological changes induced by CLP. In vivo measurement of intestinal permeability showed that CORM-2 alleviates the increase in intestinal permeability in septic rats. Moreover, inhibiting the secretion of proinflammatory cytokines and restoring the expression of TJ proteins by CORM-2 may be part of the mechanisms of the reduced permeability. Finally, we also obtained evidence that in CLP-induced septic rats, CORM-2 could suppress the MLC phosphorylation, a major regulator of intestinal epithelial permeability.

The intestinal barrier, composed of the apical cell membrane of the enterocytes and the intercellular TJs, prevents the entrance of harmful microorganisms, antigens and toxins from the lumen into the blood. Apical tight junctions are multifunctional structures form a tight seal between adjacent epithelial cells that stops para-cellular diffusion of large molecules across the epithelium [27]. Therefore, an intact TJ barrier is crucial to the function of intestinal barrier and prevention of bacterial translocation. During many illnesses, such as stress, Crohn’s disease, ulcerative colitis (UC), and polymicrobial sepsis, there is a significant alteration of intestinal permeability as the integrity of the TJ barrier becomes compromised [28–32]. Therefore targeting intestinal TJs to regulate intestinal epithelial permeability may constitute an important route for therapy. TJs are constructed by transmembrane adhesion molecules, such as the claudins, occludin and junctional adhesion molecules (JAM). The JAM intra-cellularly links to the actin filaments via various adaptor proteins, such as members of the ZO family [2]. The interaction between TJ proteins plays a crucial role in maintaining the structure of tight junctions and epithelial barrier function [33,34].

Our results showed that expression of intestinal epithelial TJ proteins was significantly reduced in the CLP-induced sepsis. Therefore, we hypothesized that increased intestinal permeability in CLP rats might be related to the decreased TJ proteins. This decrease may be due to excess secretion of inflammatory cytokines, such as TNF-α and IL-1β. It has been reported that TNF-α and IL-1β can down-regulate the expression of TJ proteins [35–37]. In this study, CORM-2 reduced serum levels of TNF-α and IL-1β in septic rats and this may be a reason for the improvement of TJ protein expression. It is known that increased expression of TJ proteins could improve intestinal epithelial barrier function [25,38] as we observed in CORM-2 treated CLP rats. This cause-effect relationship has been discussed in our previous report [39].

Myosin light chain (MLC) is a cytoskeleton protein and its phosphorylation play a significant role in the regulation of IETJs [3,40]. Increases in MLC phosphorylation levels induce the cytoskeleton to contract to increase intestinal epithelial permeability in burn mice [41]. In CLP rats, we observed a remarkable increase in phosphorylation levels of MLC from 12 h after CLP, indicating that the MLC pathway may play a critical role in mucosal barrier dysfunction during sepsis. CORM-2 inhibiting the phosphorylation of MLC suggests that CORM-2 protects the intestinal barrier, possibly through its effect on MLC phosphorylation. How CORM-2 affects MLC phosphorylation remains to be elucidated.

Tricarbonyldichlororuthenium-(II)-dimer (CORM-2) is a small chemical that has been used *in vitro* and *in vivo*, including mice and rats [17,42]. No severe side effects at 8 mg/kg i.v. have been reported. In our experiment, we have not observed any obvious side effect in CLP rats used in this study or in heathy rats injected with CORM-2 (data not shown). None of the rats lost weight or showed undesired infection. They generally stayed healthy except the ones died from sepsis. The data strongly suggest that CORM-2 has great potential to be developed as a new therapeutic reagent in the near future. This study has laid a foundation for CORM-2 to be used to improve intestinal epithelial barrier function in patients with sepsis.

In conclusion, our data demonstrate that CORM-2 is capable of improving the survival of CLP rats, reducing sepsis-induced proinflammatory cytokine release, and alleviating intestinal epithelial tight-junction damage. We speculate that CORM-2 may serve as a potential therapeutic agent to restore the normal intestinal barrier function and reduce mortality in sepsis.
